# Local proliferation is the main source of rod microglia after optic nerve transection

**DOI:** 10.1038/srep10788

**Published:** 2015-06-02

**Authors:** Ti-Fei Yuan, Yu-Xiang Liang, Bo Peng, Bin Lin, Kwok-Fai So

**Affiliations:** 1 School of Psychology, Nanjing Normal University, Nanjing, China; 2 Department of Ophthalmology, The University of Hong Kong, China; 3 State Key Laboratory of Brain and Cognitive Sciences, the University of Hong Kong, Hong Kong, China; 4 Department of Anatomy, Li Ka Shing Faculty of Medicine, The University of Hong Kong, Hong Kong, China; 5 GHM Institute of CNS Regeneration, Jinan University, Guangzhou, China

## Abstract

Microglia are the resident phagocytic cells with various functions in the central nervous system, and the morphologies of microglia imply the different stages and functions. In optical nerve transection (ONT) model in the retina, the retrograde degeneration of retinal ganglion cells (RGCs) induces microglial activations to a unique morphology termed “rod” microglia. A few studies described the “rod” microglia in the cortex and retina; however, the function and origin of “rod” microglia are largely unknown. In the present study, we firstly studied the temporal appearance of “rod” microglia after ONT, and found the “rod” microglia emerge at approximately 7 days after ONT and peak during 14 to 21 days. Interestingly, the number of “rod” microglia remarkably decays after 6 weeks. Secondly, the “rod” microglia eliminate the degenerating RGC debris by phagocytosis. Moreover, we found the major source of “rod” microgliosis is local proliferation rather than the infiltration of peripheral monocytes/hematopoietic stem cells. We for the first time described the appearance of “rod” retinal microglia following optic nerve transection.

Microglia detect injury signals in the central nervous system (CNS) and get activated. In the brain they undergo different stages of activation, which can be classified according to the morphological and immune-reactive diversities^1^,^2^. Retinal microglia represent a different population of microglial cells than brain microglia, given the distinct interaction partners and the microenvironments. Investigating retinal microglia activation and de-activation helps to understand the cellular mechanism underlying neurodegenerative diseases in the retina^3^,^4^, and therefore the relevant therapies for neuroprotection.

Optic nerve transection (ONT) is a well-established model to induce progressive retinal ganglion cell (RGC) loss, and triggers retinal microglial activation major at the ganglion cell layer (GCL)/nerve fiber layer (NFL)^5^,^6^. These activated retinal microglial cells also proliferate, and express progenitor cell markers such as nestin, Vimentin, and NG2^7^,^8^. However, their functions are still unknown. Besides, in previous studies^5^,^6^,^8^, retinal sections were routinely used for immunohistochemical staining while the global views at the GCL/NFL are usually lacking. In this study, we examined the general patterns of microglial activation and proliferation at the GCL/NFL by whole-mount immunohistochemistry.

## Materials and Methods

### Ethics

All Experiments were carried out according to the Guide of the Committee of Use of Laboratory Animals for Teaching and Research (CULATR) of The University of Hong Kong and Nanjing Normal University Animal research Ethic Committee. The study has been approved by Animal research Ethic Committee in The University of Hong Kong and Nanjing Normal University.

### Animals

40 adult male Sprague–Dawley (SD) rats (220–250 g, aged 8–10 weeks) were used in the experiments. The animals were housed with food and water *ad libitum* under 12-hour light/12-hour dark cycle (7:00 AM–7:00 PM). For the surgery, the animals were anesthetized and maintained with muscular injections of a mixture of ketamine (80 mg/kg) and xylazine (8 mg/kg). For ONT, 0.5% alcaine (Alcon-Couvreur, Puurs, Belgium) was applied to the eyes prior to the surgery, and antiseptic eye drops (Tobres [Tobramycin 0.3%]; lcon-Couvreur) were used to prevent infection after the procedures. Finadyne (0.025 mg/mL (Sigma) in drinking water was applied for 7 days to relieve the pain after the surgeries when needed. All animals were sacrificed with overdose of pentobarbital at different time points of interest.

### Optic nerve transection (ONT)

For ONT, after the animal was anesthetized by ketamine (80 mg/kg) and xylazine (8 mg/kg), the posterior pole of the eye was exposed through a superior temporal intra-orbital approach. The eyelid was lifted up using a suture, and bulbar conjunctiva was cut coronally to expose the superior extraocular muscles. By lifting up the muscles using forceps, the intraorbital portion of ON was exposed and its dura sheath was opened longitudinally. A complete transection was made to ON at 1.5 mm posterior to the optic disc as previously described. Care was taken to maintain the blood supply to the retina.

The animals were sacrificed 1, 3, 7, 14, 21 days, 6 weeks and 8 weeks after ONT, and retinas were harvested and whole-mounted for immunostaining.

### RGC retrograde labeling by *Fluoro-Gold* (FG)

Same way as ONT to expose optic nerve, a complete transection was made to the ON 1.5 mm posterior to the optic disc. A piece of gel foam soaked with 6% ***Fluoro-Gold***(FG) (Sigma, HK) was placed at the proximal optic stump. Care was taken to maintain the blood supply to the retina.

### Retinal whole-mount immunohistochemistry

The retinas were fixed by 4% PFA (Sigma) at room temperature for 1 hour, followed by PBS (Sigma) wash. Then, the retinas were blocked by 0.5% triton, 1% bovine serum albumin (BSA, Sigma) and 10% goat serum (Jackson) in PBS at room temperature for 2 hours. After that, they were incubated in a diluted primary antibody solution overnight at room temperature. After sequential PBS wash they were incubated in secondary antibody solution at room temperature for 2 hours. Finally, the retinas were washed in PBS and mounted with fluorescein mounting medium (Dako). The antibodies used included: rabbit anti-Iba1 (1:500, Wako), rabbit anti-GFP (1:500, Invitrogen), chicken anti-GFP (1:250, Invitrogen), mouse anti-GFAP (1:500, sigma), isolectin-Alexa Fluor 488/568 (1:400, Invitrogen), mouse anti-CD68 (1:500, Bio-Rad); rat anti-BrdU (1:200, Abcam), mouse anti-beta III tubulin (1:500, Covance), goat anti-mouse IgG Alexa Fluor 488/568 (1:200, Invitrogen), goat anti-rabbit IgG Alexa Fluor 488 (1:200, Invitrogen) and goat anti-rat IgG Alexa Fluor 568 (1:200, Invitrogen).

### Retinal whole mount BrdU immunohistochemistry

The animals received 4 pulses of BrdU (Invitrogen) injection on the day of interest at the dose of 50 mg/kg per injection (every 2 hours). For BrdU immunohistochemistry, retinas were first incubated in 4N HCl for 20 minutes at room temperature and then washed with 0.2 M Borate buffer (pH 8.5) for 20 minutes before another PBS wash for 5 minutes^9^. Then the retinas were processed for immunohistochemistry as described above.

### Mice parabiosis model

For mice parabiosis model, the procedures were performed as previously described^10^,^11^. Pairs of C57BL/6 wild-type (WT) mouse and Cx3cr1^+/GFP^ mouse with similar weight and same sex were used. Briefly, the flanks of animals were cleaned with a shaver and sterilized before two incisions were made on the opposite side of the two mice, from behind the ear to the hip. The non-absorbable 3-0 suture was used to join elbows and knee joints of the two animals together. Then the skins of the two animals were sutured with 5-0 absorbable suture continuously. Body temperature of mice was maintained by heating pad at 37 °C until completely recovered from anaesthesia and fed with normal water and food in a purpose-made container.

The parabiotic partners share their blood through capillary subcutaneously. 14 days after surgery the GFP positive macrophages/monocytes were found in the blood of WT mouse with flow cytometry, and also the GFP positive macrophages replenished in the spleens of WT parabionts. Then the parabiotic partners were subjected to ONT with both mice anesthetized (the partners received i.p. injections separately).

### Microscopy

The whole-mount retinas were visualized with a Zeiss Axiophot epi-fluorescence microscope; Zeiss LSM 700/710 confocal microscopes were used to collect z stacks for 3 d volumes for analysis. Both bright field and fluorescent images were collected sequentially.

## Results

### Temporal information of microglia activation

**Figure 1:**
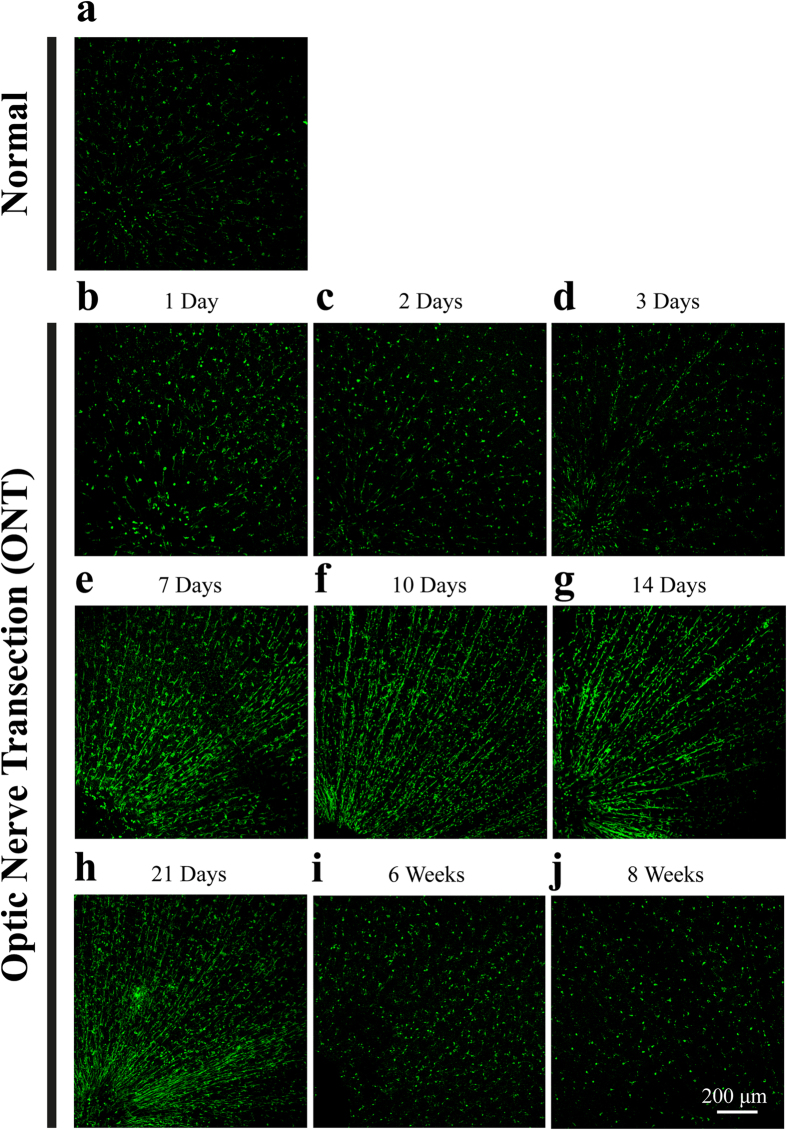
Retinal whole-mount immunostaining of microglial cells at different time points after optic nerve transection (ONT). **a**, **b**, **d**, **d**, **e**, **f**, **g**, **g**, **i** and **j** showed Iba1 staining (microglia marker) of retina under resting condition, 1 day, 2 days, 3 days, 7 days, 10 days, 14 days, 21 days, 6 weeks and 8 weeks after ONT, respectively. The microglia cells in the retina align to each other at 7 to 21 days after ONT, showing “rod” morphology; while the activation tuned down at 6 weeks after ONT. Scale bar: 200 μm.

**Figure 2:**
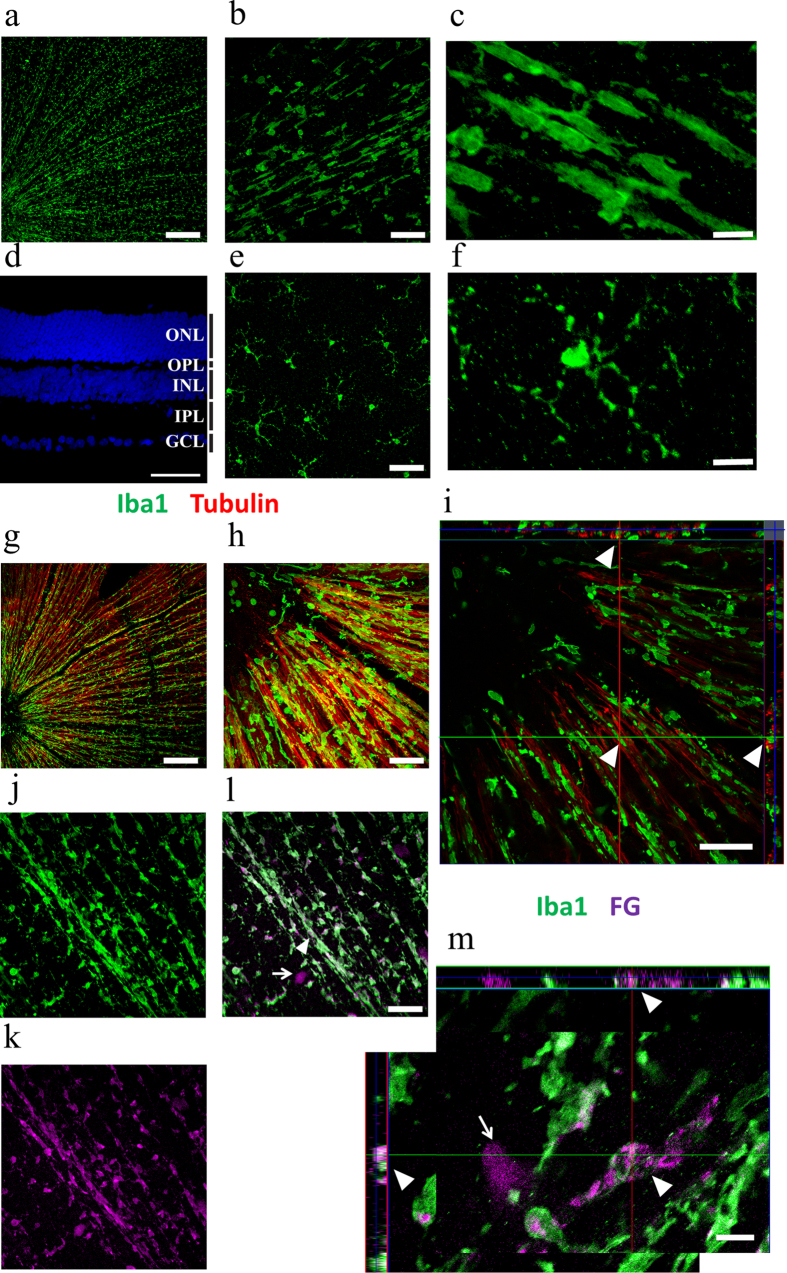
Rod microglia are specific to ganglion cell layer and are phagocytic. **a:** Whole retina staining of Iba1 3 weeks after ONT. Scale bar: 200 μm. **b**: Rod-morphology microglia at ganglion cell layer (GCL) layer of retina. Scale bar: 50 μm. **c**: High magnification image of Rod-morphology microglia at GCL. Scale bar: 10 μm. **d**: DAPI staining of retina (view from laterally) showing different cellular layers. Scale bar: 50 μm. **e**: Resting morphology microglia at Inner nuclear layer (INL). Scale bar: 50 μm. **f**: High magnification image of resting-morphology microglia at INL. Scale bar: 10 μm. **g**: Whole retina staining of Iba1 (green) and tubulin (red) 2 weeks after ONT. Scale bar: 200 μm. **h**: Close relationship between microglial processes and the nerve fibers. Scale bar: 50 μm. **i**: Z-stack view of close relationship between microglial processes and the nerve fibers (white triangle). Scale bar: 50 μm. **j, k** and **l**: Whole retina staining of Iba1 (green, 2J) and Fluoro-Gold (FG) (purple, 2K) 2 weeks after ONT. 2L is the merged picture. The arrow shows a survived ganglion cell from ONT. Scale bar: 50 μm. **m**: Z-stack view showing that FG particles were phagocytized by microglia after ONT (white triangle). Scale bar: 10 μm.

To investigate the cell morphology, we used microglia specific marker Iba1 to label the microglia^12^. At day 1 and day 3 after ONT, activated microglial cells with ameboid shape could be observed occasionally (Fig. 1b–d), compared to microglia in normal retinas (Fig. 1A, Fig. 2E,F).

Interestingly, 7 days after ONT, almost all the area of the GCL/NFL in the retinas was covered activated microglial cells, which aligned to each other and exhibited the “rod” phenotype (Fig. 1e). This phenomenon became much more evident at day 14 and day 21 after ONT, with >80% microglia cells showing “rod” morphology (Fig. 1g,h, Fig. 2a–c), which coincident with the loss of most RGCs after ONT at these time points: after proximal ONT, about 50% RGCs would be lost at day 7, which increased to 90% at day 14^13^.

At longer time points (6 weeks and 8 weeks after the cut), we found that the rod microglia disappeared and there were few fully activated microglial cells in the layer of GCL/NFL (Fig. 1i,j), which coincident with the loss of most RGCs after ONT at these time points.

### “Rod” microglia scavenge the RGC debris by phagocytosis after ONT

ONT leads to retrograde degeneration of RGCs in several days, and therefore we mainly focused on microglial cells in GCL/NFL (Fig. 2d). The “rod” microglial cells linked to each other and the pattern might follow either blood vessels or optic nerve fibers in the retina. We found that “rod” microglial cells located adjacent to nerve fibers rather than blood vessels, shown by beta-tubulin staining (Fig. 2g–i). Interestingly, there were also “round” microglial cells that are distant from the nerve fibers (Fig. 2h,i), suggesting that they might be a functionally distinct population of microglial cells.

In the CNS, microglia exert as the phagocytic immune cells during neurodegeneration. To identify whether the “rod” microglia are phagocytic, we utilized Fluoro-Gold (FG) to retrogradely label RGCs. The FG particles would be taken up by the axons of RGCs and retrogradely transported to their cell bodies. Thus, only the RGCs and their axons can be labeled with FG. We reasoned if the microglia were capable of scavenging the cell debris of RGCs, the FG particles in the RGCs would also be engulfed by phagocytosis. To test whether the “rod” microglia are capable of scavenging RGC debris after ONT, we retrogradely labeled RGCs with FG, followed by ONT a few days later. Indeed, FG was found co-localized with Iba1-positive microglia in the retinas three weeks after ONT (Fig. 2m). Moreover, the “rod” microglia were the major population of FG-containing microglia (Fig. 2m). The result suggested that “rod” microglia play an active role by scavenging RGC or RGC fiber debris after ONT.

### “Rod” microgliosis is mainly contributed by the resident microglial proliferation with limited contribution from circulating monocytes/hematopoietic stem cells (HSCs)

**Figure 3:**
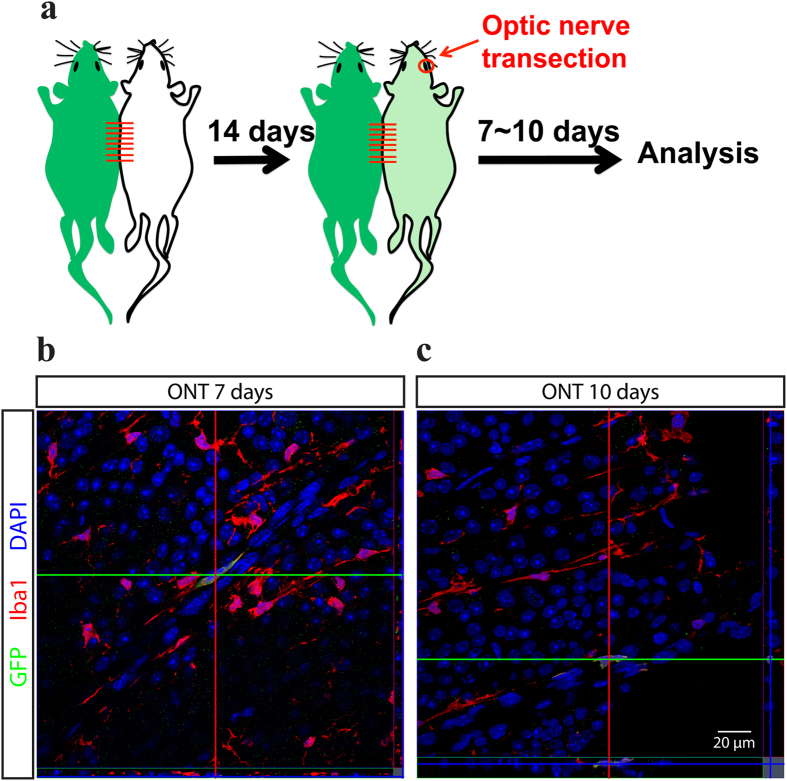
Rod microglia rarely originated from peripheral infiltrating macrophages. **a**: Pairs of C57BL/6 wild-type (WT) mouse and Cx3cr1^+/GFP^ mouse were joined for parabiosis model for at least 14 days to allow the mixed circulation. Then the ONT was performed on the WT mouse, and the tissues were harvested at 7 to 10 days after the ONT. **b**: 7 days after ONT, the mouse retina exhibited rod microglia as well, however only one cell in the view showing GFP signal. Green: GFP. Red: Iba1 staining. Blue: DAPI. Scale bar: 50 μm. **c**: 10 days after ONT, the mouse retina exhibited rod microglia as well, however only one cell in the view showing GFP signal. Green: GFP. Red: Iba1 staining. Blue: DAPI. Scale bar: 50 μm.

The number of microglia in GCL/NFL largely increased after ONT in a short period. Two pools were considered to be the major sources of microgliosis: local microglial proliferation and circulating monocytes/HSCs differentiation^14^,^15^. To study the source of “rod” microglia, we firstly utilized the parabiosis model by connecting C57BL/6 wild-type (WT) mouse with Cx3cr1^+/GFP^ mouse (Fig. 3a), in which the Cx3cr1 lineage cells including microglia, macrophages and monocytes express GFP. 14 days post-surgery, GFP-positive monocytes were found in the blood of WT parabionts by both immunocytochemistry and flow cytometry (data not shown), which demonstrates the parabiotic partners successfully share their blood. Furthermore, we examined the peripheral organs including lungs and livers, in which monocytes/HSCs differentiate into macrophages at a higher turnover rate than CNS^16^,^17^,^18^. GFP-positive macrophages were found in the lungs and livers of WT parabionts. These demonstrated our parabiosis model successfully shared the blood of the two mice, and the circulating monocytes/HSCs deriving hypothesis could be verified by observing whether there were GFP-positive macrophages/microglia. 14 days after parabiosis surgery, we performed ONT in the WT parabionts to check whether the “rod” microglia were originated from infiltrating monocytes/HSCs. Consistent with observations from rats, the mouse retinal microglial cells exhibited “rod” like morphology at 1 week after ONT. However, at all the time points we examined (7 days, 10 days and 14 days after ONT), only a minority of microglia were GFP positive in the retinas of WT parabionts (Fig. 3b,c, estimated 0.5%-1%). The results indicate that circulating monocytes/HSCs are not the major source of “rod” microgliosis.

**Figure 4:**
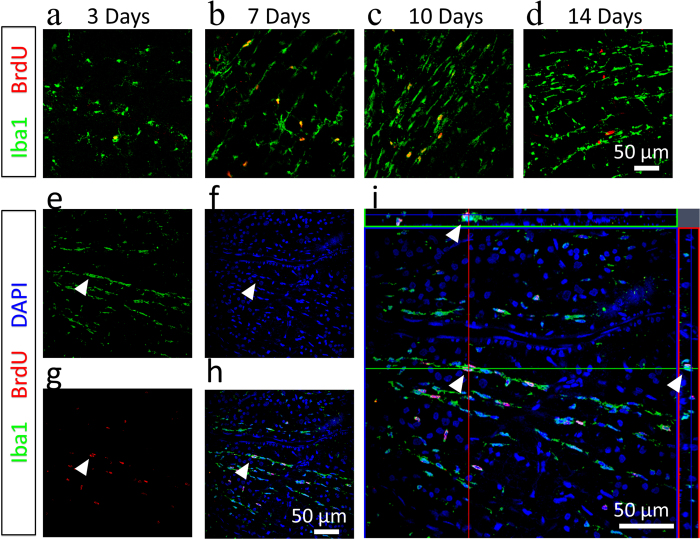
Rod microglia are proliferating *in situ*. **a, b, c** and **d**: Whole retina staining of Iba1 (Green) and BrdU (Red) at 3 days, 7 days, 10 days and 14 days after ONT. Scale bar: 50 μm. **e, f, g**, and **h**: Co-localization of BrdU (red) and Iba1 (Green) staining in a microglial cell at 7 days after ONT (white triangle). Scale bar: 50 μm. 3I: Z-stack view showing the proliferating microglia cell (white triangle). Scale bar: 50 μm.

Next we reasoned the microglial proliferation ought to be the major source of “rod” microgliosis after ONT, As in varies of models, microglial cells proliferate in response to CNS insults^19^,^20^. To this end, we injected BrdU intraperitoneally at different time points after ONT to label proliferating cells. We found that “rod” microglial cells were indeed dividing, which was evidenced by both BrdU-positive staining and DAPI-revealed nuclei (Fig. 4e–i). Notably, the proliferating rate peaked at day 7 after ONT, in compared to day 3 and day 14 (Fig. 4a–d), which was in coincidence with the RGC death curve after ONT, implying that the proliferation of “rod” microglia is triggered by local inflammation signaling. Taken together, our results indicate local cell proliferation is the major source of “rod” microgliosis after ONT, while the contribution of circulating monocytes/HSCs is limited.

## Discussion

In present study, for the first time we described the appearance of “rod” retinal microglia following ONT, which are mainly sourced from local cell proliferation. The rod-like microglia was originally discovered in cortex injury^21^, and recently demonstrated in retina in animal model of glaucoma as well^22^,^23^. A recent study set up the morphological characteristics for rod microglia in cortex, including the cell length/width ratio, retraction of secondary processes, alignment to each other and the soma polarization^24^. Interestingly, in brain injury the rod microglia appear 1-2 days after the injury^21^, while in our ONT model it is 7 days after ONT. This is consistent with the time course of ganglion cell death and axons degeneration after ONT, implying the roles of inflammation signaling in alignment and polarization of these rod microglial cells.

We have further confirmed the functions of these rod microglial cells as phagocytic cells. This suggested that these rod microglia are in their final stage, rather than acting as “transient state” cells. In fact, they lasted for several weeks in our observation, and disappeared after 2 months when RGCs death wave is completed and no debris is left. How would rod microglia return to resting state or undergo cell apoptosis after the phagocytosis is yet to be investigated. The deactivation of retinal microglia after RGC loss is critical since over-activation of microglial cells could be detrimental to the rest neurons in other layers, such as bipolar cell. Our data confirmed the spatial and temporal precision of retinal microglia response to RGC axotomy.

In brain injury, the disruption of blood-brain barrier often leads to infiltration of macrophages/monocytes into the injury site, which may contribute to the local phagocytic responses. We excluded such possibility in retina with mice model of parabiosis using the mice whose microglia, macrophages and monocytes carrying green fluorescence proteins. Our result suggested that ONT did not trigger severe disruption of blood-retinal barrier and peripheral macrophages could not penetrate into the retina after injury in present model. It is, however, possible that direct disruption of the vascular system in retina, such as hemorrhage or ischemia, could result in peripheral macrophage infiltration to the eye.

Our present study is however limited to morphological observations. It will be interesting to investigate if these rod microglial cells are functionally coupled to each other with gap junctions^25^,^26^. Such possibility allows these cells to respond to distal injury signals and reorganize the cytoskeleton for repolarization, through calcium wave, for instance. It will also be useful to examine the lineage of each rod microglia – to see if the coupled cells are originated from one grandmother “stem-like” microglia. Notably, these microglia cells divide while maintain complex morphology – implying the importance of environmental signals in regulating microglia proliferation.

In summary, we have characterized the retinal microglia responses to ONT by whole mount immunostaining. The newly added classification of rod microglia into retinal microglia provided more knowledge in the morphological diversity and functional variances of microglial cells in the nervous system.

## Additional Information

**How to cite this article**: Yuan, T.-F. *et al.* Local proliferation is the main source of rod microglia after optic nerve transection. *Sci. Rep.* **5**, 10788; doi: 10.1038/srep10788 (2015).
